# Characterisation of a large, single-centre cohort of patients with Becker muscular dystrophy to inform standardised care guidelines

**DOI:** 10.1007/s00415-025-13126-9

**Published:** 2025-06-07

**Authors:** Pietro Riguzzi, Holly Borland, Meredith K. James, John Bourke, Chiara Marini Bettolo, Robert Muni Lofra, Jordi Diaz-Manera, Giorgio Tasca, Marianela Schiava, Maha ElSeed, Elizabeth Harris, Emma Grover, Chloe Geagan, Carla Bolano Diaz, Ariele Barreto Haagsma, Doaa Salman, Tara Reeves, Goknur S. Kocak, Emma Robinson, Peter Waldock, Michelle McCallum, Jassi Michell-Sodhi, Dionne Moat, Karen Wong, Ana Topf, Elena Pegoraro, Luca Bello, Volker Straub, Michela Guglieri

**Affiliations:** 1 John Walton Muscular Dystrophy Research Centre, Translational and Clinical Research Institute, Newcastle University and Newcastle Hospitals NHS Foundation Trust, Central Parkway, Newcastle Upon Tyne, NE1 3BZ UK; 2 Department of Neurosciences DNS, University of Padova, Padua, Italy

**Keywords:** Becker muscular dystrophy, BMD, dystrophinopathy, muscular dystrophy, dystrophin gene, DMD

## Abstract

**Aims:**

This retrospective, cross-sectional study aimed to characterise a large cohort of paediatric and adult patients with Becker muscular dystrophy (BMD) to inform clinical care.

**Results:**

The analysis included data from 163 male patients with genetically confirmed BMD followed up at a highly specialised neuromuscular centre between 1982 and 2023. The mean age at last neuromuscular assessment was 33.2 years (range 1.4–86.3). Large deletions in the *DMD* gene were the most common variants (78% of cases), followed by large duplications and small variants, each accounting for 11% of cases. BMD diagnosis was prompted by skeletal muscle symptoms in 52.2% of cases, a positive family history in 27.6%, neuropsychiatric issues or diagnoses in 9.7%, incidental findings in 6.7%, and cardiomyopathy in 3.8%. Twenty-three percent of patients were non-ambulant at last evaluation, with a mean age at loss of ambulation (LoA) of 42.2 years (range 11.2–77.6 years). Disease duration correlated with the severity of motor impairment (expressed as fully ambulant, ambulant with limitation, ambulant with aids, non-ambulant) at last assessment. Cardiac involvement was observed in 52.3% of patients. Severe respiratory impairment was rare and more prevalent in non-ambulant patients. Neuropsychiatric issues were common (44.2%), but only 18.4% of patients had a formal diagnosis.

**Conclusions:**

Retrospective analyses of clinical case records contribute to improved understanding of the variability of phenotypes of BMD. Combined with data from other large cohorts, these findings can contribute to the development of standard of care guidelines for BMD and inform the design of clinical trials of novel therapies.

**Supplementary Information:**

The online version contains supplementary material available at 10.1007/s00415-025-13126-9.

## Introduction

Becker muscular dystrophy (BMD) is an X-linked recessive neuromuscular disorder characterised by progressive muscle weakness and wasting [1]. Unlike the more severe, allelic form, Duchenne muscular dystrophy (DMD), in BMD, in-frame variants in the *DMD* gene allow the production of a partially functional dystrophin protein [2]. Large deletions account for approximately 78% of cases, followed in frequency by large duplications, small insertions or deletions, missense, synonymous, or intronic variants [2]. Nonsense and frameshift variants can rarely also result in some production of dystrophin and a BMD phenotype [3–5].

There is a high degree of phenotypic variability in BMD, although muscle weakness and wasting are predominant in most patients. The age of onset and severity can vary significantly, ranging from a DMD-like phenotype, with childhood-onset, severe progression and early loss of ambulation (LoA), to late adult-onset forms with mildly progressive limb-girdle weakness [1, 6]. Some affected individuals can remain asymptomatic but with persistently high serum creatine kinase (CK) levels, or report myalgias and cramps, with or without recurrent episodes of rhabdomyolysis and/or myoglobinuria [1, 6–8]. Cardiac involvement is common, but its severity can range from having subtle signs of non-ischaemic fibrosis on cardiac magnetic resonance imaging (cMRI) to severe dilated cardiomyopathy and heart failure [9–11]. Cardiac involvement can occur in isolation or in association with skeletal muscle symptoms; it can be the presenting feature or manifest at any stage of the disease. Severe respiratory impairment is rare, typically only occurring at later stages of the disease and in non-ambulant patients [12, 13]. Finally, neuropsychiatric features, including intellectual disability and autism, have been reported in BMD [14–16].

Currently, there are no specific therapies for BMD. Based on the experience in DMD, corticosteroids may be prescribed for patients with more severe skeletal muscle phenotypes [17]. However, the lack of strong evidence of the benefits of corticosteroids in BMD, combined with the well-known risks of long-term steroid use, means that corticosteroids are not routinely used in BMD. Cardioactive medications are prescribed when cardiac imaging shows evidence of segmental or global left ventricular (LV) dysfunction. However, unlike for patients with DMD, cardioprotection is not currently advocated prophylactically. Because of the milder overall phenotype and longer survival in many patients with BMD, cardioverter-defibrillator implantation, left ventricular assist device, and cardiac transplantation are more often considered than for patients with DMD. However, in the absence of supporting evidence or BMD care guidelines, these options are only invoked on a case-by-case basis with significant variation among different centres and different countries [18, 19].

Compared to the extensive knowledge accrued on the natural history and rate of progression of DMD, our understanding of the phenotypic variability and rates of progression in patients with BMD so far has been based on data from a few relatively small studies [6, 8, 20, 21]. While more recently data from larger multi-centre studies have contributed to better characterise clinical presentations and progression in BMD [22–24], there is still a gap in knowledge to support the development of internationally agreed guidelines for the diagnosis, management, or treatment of patients with BMD.

Here we describe the clinical presentation of a large cohort of patients with BMD followed up at a highly specialised centre for neuromuscular diseases. The monocentric nature of this study ensures robust and consistent data collection and can contribute to provide valuable guidance for the development of standardised care protocols.

## Methodology

This is an observational, retrospective study with a cross-sectional design based on extraction of clinical and genetic data from a large cohort of paediatric and adult patients with a confirmed diagnosis of BMD followed at the John Walton Muscular Dystrophy Research Centre (JWMDRC) in Newcastle upon Tyne, United Kingdom (UK), from 1982 to 2023. Data, amassed from routine neuromuscular clinic attendances, included information on molecular diagnosis; age and nature of first concerns leading to the diagnosis; key motor function milestones; cardiac and respiratory function; neuropsychiatric and psychiatric co-morbidities.

The nature of first concern leading to a diagnosis of BMD was categorised as family history, incidental finding, muscle signs/symptoms, cardiac signs/symptoms, and neuropsychiatric issues or diagnoses. Incidental findings comprised incidental CK measurements showing hyperCKaemia or genetic investigations conducted because of the suspicion of other clinical conditions, which led to the diagnosis of BMD. Muscle signs and symptoms encompassed delayed motor milestones; muscle pain and cramps; rhabdomyolysis and myoglobinuria; and signs of muscle weakness (e.g., poor sport performance, gait abnormalities, frequent falls, difficulties in rising from the floor or climbing stairs). Those presenting with cardiac signs/symptoms as their first concern were diagnosed with BMD following detection of cardiomyopathy on cardiac imaging (echocardiogram and/or cMRI). Neuropsychiatric issues included behavioural problems, delayed speech development, learning difficulties, and other neurodevelopmental features, with no formal diagnosis, as reported by clinicians in clinical notes in the paediatric phase. Neuropsychiatric diagnoses included formal diagnoses of autism, attention deficit hyperactivity disorder (ADHD), and intellectual disability/specific learning disorders. Psychiatric disorders were also recorded and included psychiatric symptoms (e.g. anxiety, low mood) and psychiatric diagnoses (e.g. bipolar disorder, depression).

The Diagnostic and Statistical Manual of Mental Disorders, Third Edition (DSM-III) was published in the 1980s and, for the first time, provided diagnostic criteria and categorisation of neurocognitive disorders (e.g., autism and ADHD). A revised document (DSM-III-R) was published in 1987 and implemented over subsequent years [25, 26]. Therefore, to take into account the changes in these diagnostic criteria, the prevalence of neuropsychiatric issues and diagnoses, as well as psychiatric disorders, was compared between patients who were born before and after January 1990.

Motor function was described as:

1. Fully ambulant: individuals able to walk independently, without the use of aids, and with no limitations in walking distance.
2. Ambulant with limitation: individuals able to walk independently, without the use of aids, but with significantly restricted walking distances.
3. Ambulant with aids: individuals able to walk only with the assistance of crutches, a walking stick, assistance of another person, and individuals requiring part-time use of a wheelchair.
4. Non-ambulant: individuals requiring use of a wheelchair full-time.

Cardiac involvement was defined based on echocardiography finding of left ventricular ejection fraction (LVEF) less than or equal to 50%, and/or fractional shortening (FS) less than 27%, and/or LV segmental hypokinesia, and/or evidence of a non-ischaemic distribution of myocardial fibrosis on cMRI [11, 27].

Respiratory function was assessed by measuring Forced Vital Capacity (FVC) in a sitting position and was expressed as percentage predicted for age, sex, and height. FVC was categorised as [28, 29]:

1. Normal: FVC ≥ 80%
2. Moderately impaired: FVC 60–79%
3. Severely impaired: FVC < 60%

FVC values were obtained by trained neuromuscular physiotherapists using standardised spirometry. To ensure reliability of FVC measures, respiratory function was only analysed for patients above the age of 10 years.

### Inclusion criteria

Patients were included based on availability of clinical data and a confirmed molecular diagnosis of BMD, defined as detection of large in-frame deletions/duplications (involving one or more exons), small deletions/duplications*,* or splice-site variants predicted to maintain the reading frame in the *DMD* gene. Patients with predicted frameshift or nonsense variants, or uncertain reading-frame retention, were included only if a muscle biopsy demonstrated dystrophin expression on Western blot and/or immunohistochemistry/immunofluorescence consistent with BMD. Individuals only exhibiting traces of dystrophin or patterns suggestive of revertant fibres were excluded. Similarly, patients carrying likely pathogenic variants or variants of unknown significance in the *DMD* gene, according to the American College of Medical Genetics and Genomics (ACMG) standards, were included, only if disease causality was supported by a consistent BMD phenotype and in silico analyses and/or dystrophin expression on muscle biopsy and/or cDNA analysis of the transcript were compatible with BMD [30].

In addition, a few patients without a genetic report were included and considered to harbour the family variant if there was a family history of BMD (one relative on maternal side with a genetically confirmed in-frame variant in the *DMD* gene), a consistent clinical phenotype, and high CK levels.

### Genetic diagnosis

Molecular diagnosis was confirmed by a range of techniques, reflecting changes in diagnostic practice over the study time span. These included multiplex polymerase chain reaction (PCR), multiplex ligation-dependent probe amplification (MLPA), Sanger, or next generation sequencing (NGS) of the *DMD* gene, or cDNA analysis of the transcript from a muscle biopsy.

### Definitions

Diagnostic delay was defined as the time between first concerns and confirmation of genetic diagnosis. As genetic testing through MLPA, *DMD* gene sequencing, and cDNA analysis of the transcript became more widely available at the end of the 1990s, only patients with onset (reported first concern) after 1 January 2000 were included in the analysis of the diagnostic delay. Disease duration was defined as difference between age at last assessment and age at first concerns. Corticosteroid use was defined as continuous treatment with oral corticosteroids for BMD.

### Statistical analysis

Descriptive statistics were used to summarise categorical variables using numbers and percentages. Continuous variables were presented with mean or median, standard error, and value ranges, as appropriate. Analysis of variance (ANOVA) was conducted to assess differences in means between groups. Spearman’s rank correlation coefficient was calculated to determine correlations between age and percentage predicted FVC values. Pearson’s correlation test was used to assess the relationship between age and CK levels, which were transformed into a base-10 logarithmic scale. An ordinal logistic regression analysis was conducted to examine genotype–phenotype associations between specific variants of the *DMD* gene and clinical severity at the most recent assessment, adjusted for patient age, as well as to explore associations between disease duration and motor milestone at last assessment. Time-to-event analysis was performed to describe disease progression through motor function milestones. Additionally, Cox regression analysis was employed to examine risk correlations between the time-to-event analysis of LoA and respiratory function (transformed into ordinal variables: 0 = normal, 1 = moderately impaired, 2 = severely impaired), as well as cardiac involvement. Fisher’s exact test was used to explore differences in the prevalence of neuropsychiatric and psychiatric disorders in patients who were born before and after 01/01/1990. Differences were considered statistically significant at the 5% level (i.e., *p* < 0.05). All analyses and summary graphs were performed using R v. 4.2.3.

### Ethical considerations

The study was approved as a service review under the Newcastle upon Tyne Trust Hospital because the project was to evaluate the current natural history data to inform decision-making process on care management for patients with BMD.

## Results

### Demographics

One hundred sixty-three patients met the inclusion criteria. Sixty-three patients were unrelated, and 100 were distributed across 36 BMD families. The follow-up period spanned from 1982 to 2023, and birth year of included patients ranged from 1926 to 2020. The mean age at the last neuromuscular assessment was 33.2 ± 20.3 years (range 1.4–86.3), and the mean duration of follow-up was 13.7 ± 9.9 years (range 0–37.1). The demographics of the cohort are summarised in Table 1.

**Table 1:** Demographics and disease milestones data

Total cohort	*n*	163
**Demographics, mean SD (min–max**)		
**Age at last neuromuscular assessment**	162/163	33.2 ± 20.3 (1.4–86.3)
Fully ambulant	58/162	18.3 ± 12.9 (1.4–62.8)
Ambulant with limitation	33/162	30.3 ± 12.5 (5.1–63.3)
Ambulant with aids	33/162	40.4 ± 18.0 (9.1–70.3)
Non-ambulant	38/162	52.4 ± 18.8 (11.5–86.3)
Age at LoA	36/38	42.2 ± 17.0 (11.2–77.6)
**Age at first concerns (excluding family history)**	95/97	11.4 ± 12.3 (0–51)
Muscle signs/symptoms	69/70	12.0 ± 12.2 (0.8–51)
Neuropsychiatric issues/diagnoses	12/13	4 ± 2.2 (2–10)
Incidental findings	9/9	4.0 ± 3.6 (0–10)
Cardiac signs/symptoms	5/5	33.8 ± 9.1 (24–46)
** Age at last respiratory assessment**	139/164	36.4 ± 19.4 (10.4–86.3)
FVC		
≥80%	89/139	31.1 ± 17.6 (10.4–84.7)
60–79%	32/139	40.2 ± 17.2 (11.4–69.1)
<60%	18/139	55.9 ± 18.5 (25.2–86.3)
**Cardiac function data**	153/163	
Cardiac involvement	80/153	
Age at detection of cardiac involvement	77/80	33.1 ± 15.7 (11.1–75.3)
Age at last cardiac follow-up	149/163	32.9 ± 20.1 (1–84.9)

*N* number, *SD* standard deviation, *LoA* loss of ambulation, *FVC* forced vital capacity

### Genetic variant analysis

The most prevalent variants were in-frame deletions involving one or more exons (large deletions), accounting for 78% (127/163) of cases. The most frequent was the deletion of exons 45–47 (41.1%, 67/163). Large deletions predominantly clustered within the mutational hotspot between exons 45–55 (92%) [2]. A comprehensive list of the *DMD* gene variants in the cohort is provided in supplementary materials (Supplementary Table 1 and Supplementary Table 2).

### Age and nature of first concern

Details of the first clinical concern leading to a diagnosis of BMD was available for 134/163 (82.2%) patients. Excluding subjects who were diagnosed because of a family history of BMD (27.6%, 37/134), mean age at first concern was 11.4 ± 12.3 years (range 0–51; Table 1). Muscle signs/symptoms were reported most commonly, by 52.2% (70/134) of patients, at a mean age of 12.0 ± 12.2 years (range 0.8–51). Neuropsychiatric issues/diagnoses were the next most common concerns reported at onset in 9.7% (13/134) of patients at a mean age of 4 ± 2.2 years (range 2–10). In the majority of these cases (8/13), the diagnosis of BMD was reached through DNA microarray analysis, which was part of local protocols used to investigate neurodevelopmental disorders in children. Incidental finding of high CK or a molecular diagnosis made while under investigation for other conditions led to a BMD diagnosis in seven and two patients, respectively (total 6.7%, 9/134), at a mean age of 4.0 ± 3.6 years (range 0–10). Cardiac signs/symptoms were the initial concerns in only 3.8% (5/134) of the cohort and presented at a mean age of 33.8 ± 9.1 years (range 24–46).

### Diagnostic delay

The following patients were excluded from the analysis of the diagnostic delay: patients for whom family history was the first concern, patients with first reported concerns before January 2000, patients without a genetic report allowing for determination of time to diagnosis, and patients with an incidental diagnosis of BMD through molecular investigations for alternative diagnoses. For the remaining patients (53/134), the mean diagnostic delay was 2.7 ± 3.0 years (range 0.1–12.4). The diagnostic delay based on nature of first concern is summarised in Table 2. No statistically significant differences were noted in the diagnostic delay based on nature of first concerns (ANOVA test *p* = 0.646).

**Table 2:** Diagnostic delay in the subgroup of patients born after 1/1/2000

Nature of first concern	Number of patients (*N*)	Mean diagnostic delay (years ± SD)	Median diagnostic delay (years)	Number (%) of patients*
Incidental high CK finding	6	1.2 ± 1.8	0.7	11.3
Muscle signs/symptoms	31	2.9 ± 2.9	1.7	58.5
Cardiac signs/symptoms	5	3.3 ± 4.4	1.4	9.4
Neuropsychiatric issues/diagnoses	11	2.7 ± 3.2	0.8	20.8

*Percentage was calculated based on the total number of patients with first concern after January 2000, excluding family history, incidental molecular diagnosis and one patient whose genetic report was not available (53/134)

### Corticosteroid use

Of the 163 patients included, only nine (5.5%) were taking corticosteroids at any point, and all were on prednisolone. Age at corticosteroid initiation ranged from eight to 33 years. The mean initiation dosage (information available for 8/9 subjects) was 29.4 ± 6.8 mg (range 25–45). Seven patients were started on a daily regimen, while two on an intermittent schedule.

Only three patients (1.8%) were still taking corticosteroids at their last assessment.

Mean duration of treatment in those who had discontinued corticosteroids was 3.2 ± 3.6 years (range 0.3–8.7). Reasons for discontinuation included lack of benefit, weight gain, depression, and diagnostic confirmation of BMD (in a subject previously suspected to have DMD).

### Motor function

At last neuromuscular assessment, 35.8% (58/162) of patients were fully ambulant, 20.4% (33/162) were ambulant with limitation, 20.4% (33/162) were ambulant with aids, and 23.4% (38/162) were non-ambulant (Table 1).

Mean age at LoA (information available for 36/38 patients) was 42.2 ± 17.0 years (range 11.2–77.6).

Of the 38 non-ambulant patients, 18 reported muscle symptoms as their initial concern. Mean time from symptom onset to LoA (17/18) was 26.4 ± 14.3 years (range 8–57.6).

All non-ambulant individuals lost ambulation after the age of 18 years, except one subject, who lost ambulation at the age of 11.2 years following a fracture.

**Fig. 1:**
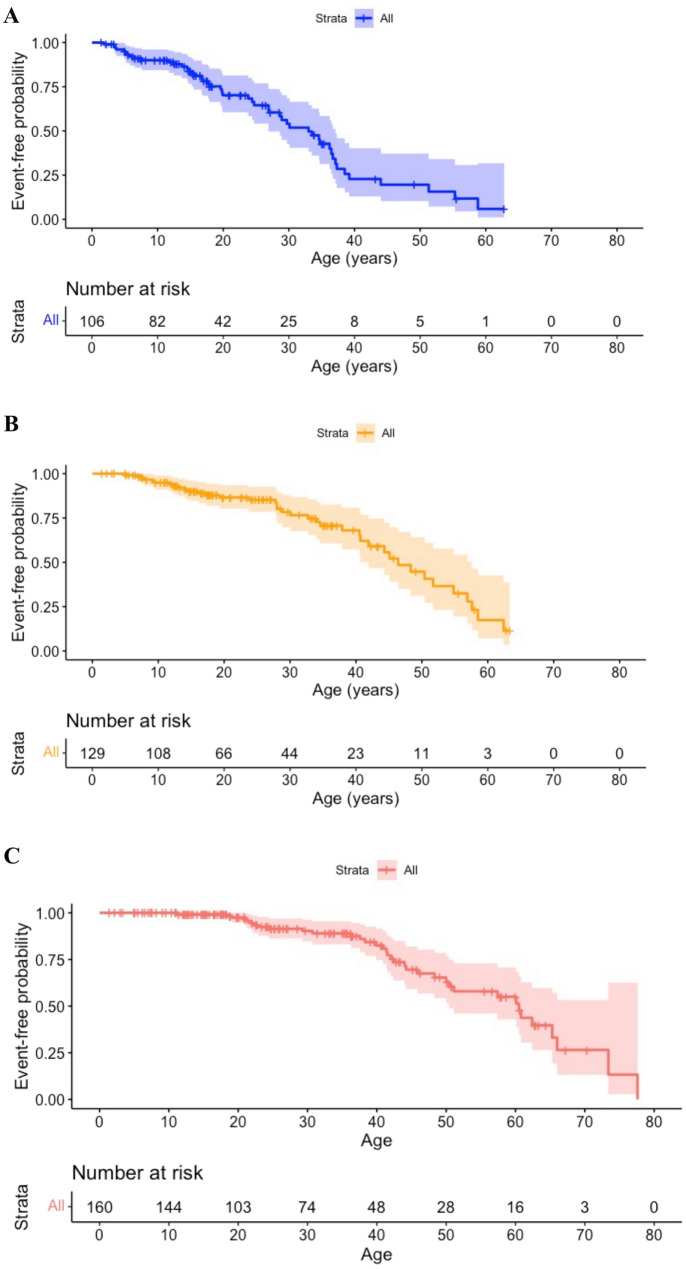
Time-to-event analysis for each disease motor function milestone. **A** time-to-event analysis of ambulant with limitation milestone. Event-free-probability: probability of walking without any limitations. *N* = 106, events = 48, median “survival” age 33 (95% CI 26.9, 37.1). **B** time-to-event analysis of ambulant with aids milestone. Event-free probability: probability of retaining ambulation either with no limitations or with limitations but without aids. *N* = 129, events = 38, median “survival” age 46.4 years (95% CI 41.9, 57.6). **C** time-to-event analysis of non-ambulant milestone (LoA). Event-free probability: probability of retaining ambulation. *N* = 160, events 36, median “survival” age 60.5 years (95% CI 50.4, NA)

Time-to-event analysis showed a median age to reach the ambulant with limitation milestone of 33.0 years (Fig. 1A), the ambulant with aids milestone of 46.4 years (Fig. 1B), and the non-ambulant milestone of 60.5 years (Fig. 1C).

### Association between disease onset, disease duration and motor function milestones

A Cox regression analysis was conducted to evaluate the time-to-event association between disease motor function milestones and age at first concerns. Patients who reported family history and incidental finding as first concerns were excluded from this analysis. A later age at first concerns was associated with a reduced risk of all disease motor function milestone events (Table 3).

**Table 3:** Cox regression analysis between disease motor function milestones’ time-to-event analysis and age at first concerns. HR: Hazard Ratio, CI (confidence interval)

Time to disease motor function milestones	HR	95% CI	*p* value
Ambulant with limitation	0.8979	0.8357, 0.9647	< 0.01
Ambulant with aids	0.90515	0.8454, 0.9691	< 0.01
Non-ambulant	0.94331	0.9001, 0.9886	0.0148

Moreover, an ordinal logistic regression was utilised to explore the association between disease motor function milestones (from fully ambulant to non-ambulant) at last assessment and disease duration. The reason of first concern was included as covariate. A significant association was observed between disease duration and severity of disease motor function milestones at last assessment (OR 1.116; 95% CI 1.068, 1.1167; *p* < 0001). When looking at the nature of first concern, subjects presenting with neuropsychiatric issues were less likely to have any impairment in ambulation compared to individual presenting with either skeletal muscle or cardiac phenotype at onset (OR 0.094; 95% CI 0.017, 0.509; *p* = 0.006).

### Genotype–phenotype associations

An ordinal logistic regression was used to explore the association between major genetic variant subgroups and disease motor function milestones at the last assessment, adjusted for age. The genetic variant subgroups included the most frequent deletions of exons 45–47 (*n* = 66, set as the reference category) versus 45–48 (*n* = 20); 45–49 (*n* = 7); 45–53 (*n* = 11); large duplications or deletions affecting regions coding for the N-terminal domain of dystrophin (from exon 2 to 8) (*n* = 7); other variants (*n* = 40); large deletions known to be associated with milder phenotypes (deletions of exons 48, 48–51, 49–51, 45–55) [21] (*n* = 11). The analysis confirmed that patients in the latter group had significantly lower odds of experiencing severe disease motor function milestones compared to those with the most frequent genotype (OR 0.203; 95% CI 0.046, 0.908;* p* = 0.037; ordinal logistic regression model Likelihood Ratio Chi-Square 96.0535, *p* < 0.0001).

### Creatine kinase

Creatine kinase levels were available for 95 of the 163 patients. For patients with multiple measurements, the value obtained closest to the onset of symptoms was used for analysis. The mean age at the time the CK was measured was 16.4 ± 17.3 years (range 0–62.8). The mean CK level was 9105.5 ± 31,453.1 U/L (median 2544; range 137–264,110). A negative correlation was found between CK levels (transformed into base 10 logarithmic scale, Supplementary Fig. 1) and age at time of CK measurement (Pearson’s test *R* = − 0.36; *p* = 0.0004).

Seven out of 95 patients (7.4%) in our cohort had CK levels below the threshold for pauci or asymptomatic hyperCKemia in non-black males (504 U/L) [31]. Among them, four had no or only mild skeletal muscle symptoms at the last assessment (mean age 23.5 ± 26.5 years, range 5.5–62.8), and were able to walk with no restrictions. Their diagnosis was prompted by factors unrelated to muscle signs or symptoms.

### Respiratory function

**Fig. 2:**
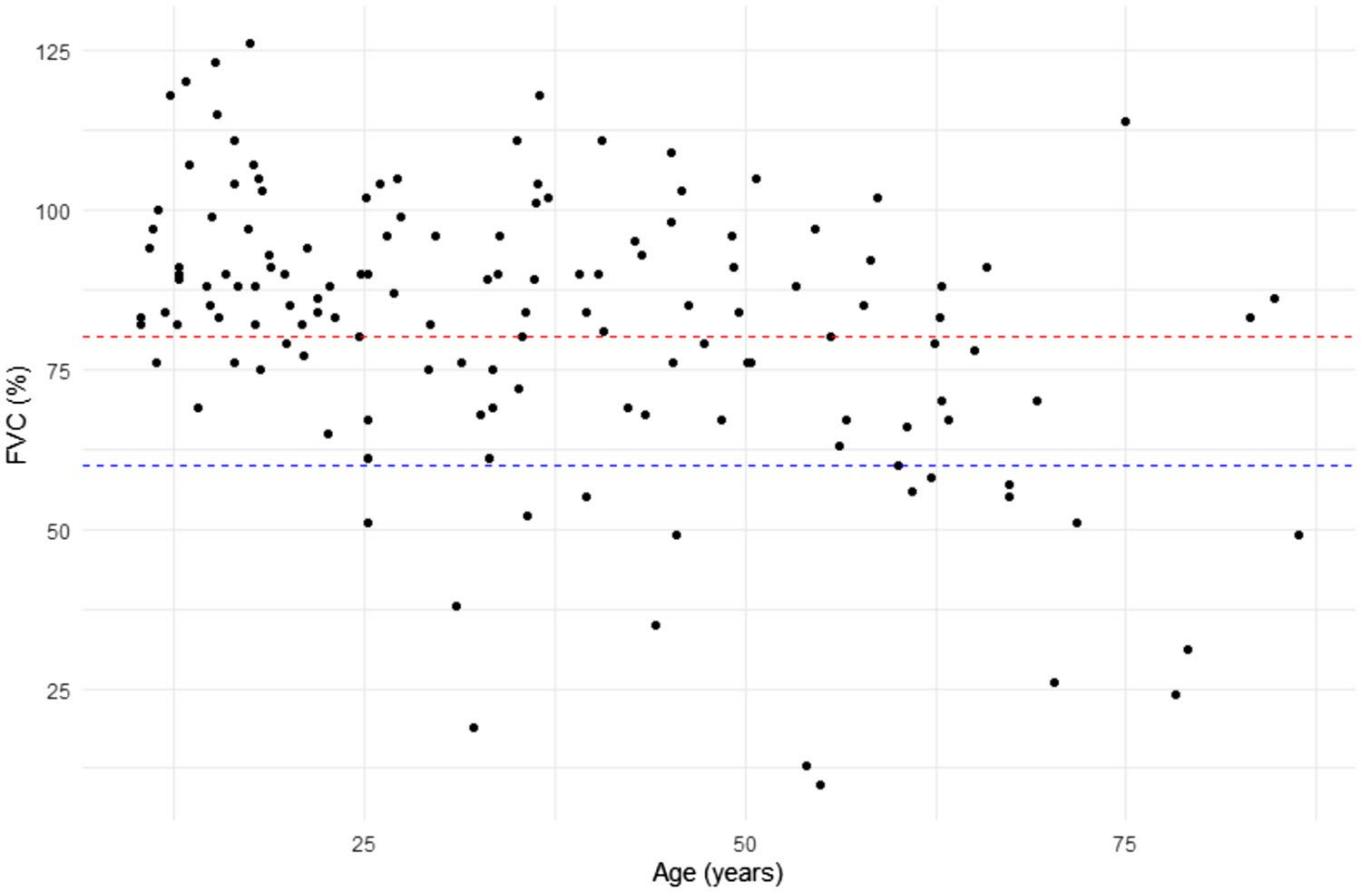
Correlation between age (years) and FVC (% predicted) at last assessment. Dashed red line represents FVC threshold of 80%; dashed blue line represents FVC threshold of 60%

An FVC measurement at last assessment was available for 139 patients (85.3%) (Table 1). The majority (64%, 89/139) had normal respiratory function, 23% (32/139) had moderately impaired respiratory function, and 13% (18/139) had severely impaired respiratory function. The majority of patients (72.2%, 13/18) with severely impaired respiratory function were non-ambulant. There was a significant negative association between FVC values (% predicted) and age at last respiratory assessment (*ρ* = − 0.371; 95% CI − 0.51, − 0.22; *p* < 0.0001) (Fig. 2).

Fifteen patients used part-time non-invasive ventilatory support (NIV); ten (66.7%) of these were non-ambulant. The mean age at NIV initiation was 53.4 ± 15.3 years (range 33.4–85.9). Only 50% of the patients on NIV had FVC values below 60% of predicted at the time of NIV initiation.

### Cardiac involvement

Measures of cardiac function were available for 153 (93.9%) patients (Table 1). The mean age at last cardiac assessment was 32.9 ± 20.1 years (range 1–84.9). Cardiac involvement was reported in 52.3% (80/153) of these patients. Age at first detection of cardiomyopathy ranged from 11.1 to 75.3 years (mean 33.1 ± 15.7 years). Within our cohort, eight patients had a pacemaker inserted, eleven had an implantable cardioverter defibrillator (ICD), four had a left ventricular assist device placement, and seven had undergone a cardiac transplant.

### Association between LoA, respiratory function, and cardiac involvement

There was a significant negative association between respiratory function categories and risk of LoA (HR = 1.7361; 95% CI 1.123639, 2.682545; *p* = 0.013), indicating a significant association between lower FVC values and higher risk of occurrence of LoA. Conversely, no significant association was found between evidence of cardiac involvement and LoA (HR 1.02; *p* = 0.966).

### Neuropsychiatric issues/diagnoses and psychiatric disorders

Of 163 patients, 72 (44.2%) were reported to have neuropsychiatric issues in childhood, including behavioural problems, learning difficulties, and/or delayed speech development. However, only 30 (18.4%) had a formal assessment and diagnosis. Among those with a formal diagnosis, 10.4% (17/163) had confirmed autism and 4.3% (7/163) had ADHD. The remaining 3.7% (6/163) were diagnosed with isolated intellectual disability or specific learning disorders (e.g., dyslexia and/or dyspraxia).

Fifty-seven patients (34.9%) had either a formal psychiatric diagnosis or significant, self-reported psychiatric symptoms. Emotional disturbances were commonest, with 28.8% (47/163) reporting depression, low mood, anxiety, and/or panic attacks. Ten (6.1%) additional patients reported substance misuse, a psychotic episode, suicidal behaviour, post-traumatic stress disorder (PTSD), bipolar disorder, or unspecified personality disorder.

The number of patients with a formal neuropsychiatric diagnosis was significantly higher among those born after 01/01/1990 compared to those born before (Fisher’s exact test *p* < 0.0001) (Table 4). In addition, 35.1% (27/77) of patients born after 01/01/1990 were reported to have neuropsychiatric issues with no official diagnosis documented compared to 17.4% (15/86) in the cohort born before 01/01/1990. The opposite trend is true for those with psychiatric diagnoses and symptoms (e.g., anxiety, depression, and low mood). Significantly more patients born before 01/01/1990 had a formal psychiatric diagnosis or reported psychiatric symptoms than those born after 01/01/1990 (Fisher’s exact test *p* < 0.01).

**Table 4:** Distribution of neuropsychiatric issues/diagnoses and psychiatric disorders within our cohort stratified by patients born before and after 01/01/1990

	Born before 01/01/1990	Born after 01/01/1990
**Neuropsychiatric issues/diagnoses**		
ADHD	1/86	6/77
Autism	1/86	16/77
Intellectual disability/specific learning disorders	2/86	4/77
No formal diagnosis	15/86	27/77
Psychiatric disorders*	39/86	18/77

*ADHD* attention deficit hyperactivity disorder

*Psychiatric disorders encompassed both diagnosed conditions and reported symptoms. Some individuals categorized under “psychiatric disorders” may have received a formal neuropsychiatric diagnosis or experienced neuropsychiatric issues during infancy

## Discussion

BMD is a condition characterised primarily by progressive muscle wasting and weakness. However, it is well recognised that there is a wide inter-patient variability in age of onset, rate of progression, and clinical manifestations [6, 8, 32]. The aims of this study were to describe this variability based on a large cohort of patients with BMD and explore factors that might explain these phenotypic differences.

Age of onset significantly varied depending on the system affected. When present, neuropsychiatric features were evident at a younger age compared to age of onset in those presenting with skeletal muscle involvement. Conversely, patients with a predominant cardiac phenotype presented later. This might be explained by the fact that cardiac manifestations typically have a long pre-symptomatic phase before patients experience symptoms of left ventricular insufficiency or arrhythmias [9, 11, 33].

As expected from the progressive nature of BMD, there were significant differences in disease duration between those who were still ambulant and those who had lost ambulation.

All non-ambulant patients, except one, lost ambulation after 18 years of age, indicating that the historically used clinical threshold of 16 years for LoA to define BMD may need to be reconsidered [1]. This is particularly relevant in the context of delayed LoA observed in DMD due to improved standards of care, as well as the potential impact of emerging disease-modifying treatments [34]. The mean age of LoA in our cohort aligns with the recent literature [22] but was higher compared to the age reported in a previous study of a BMD cohort from the same neuromuscular centre in 1993 (i.e., 42.2 years versus 37.6 years) [6]. Notably, both studies used the same definition of LoA [6]. The change in mean age at LoA may be due to improvements in multi-disciplinary care, as well as increased awareness of the variable phenotypes of BMD leading to earlier diagnosis of those with milder phenotypes than in the past.

For example, individuals carrying deletions of exons 48, 45–55, 48–51, and 49–51, previously reported predicting milder phenotypes [21, 22, 24], showed slower disease progression compared to those with the most frequent gene variant in BMD (deletion of exons 45–47). This might need to be taken into account for clinical trial study design and interpretation of the impact of new investigational drugs. Interestingly, these ‘milder’ variants were found less frequently in our cohort compared to in other studies. This may be due to lack of routine CK level testing in the UK, which might result in under-detection of BMD in pauci-symptomatic individuals [23]. It is also worth highlighting that CK levels did not meet the threshold of abnormality recommended by the European Federation of Neurological Societies (EFNS) in some asymptomatic or mildly symptomatic patients in our cohort [31]. A lower reference threshold of abnormality may be more appropriate when evaluating patients for a potential diagnosis of BMD. The use of CK as a biomarker for disease progression remains debatable; in fact, in our cohort, we observed that when CK levels were within normal limits, this was independent of the patient’s age and motor function.

In our study, the mean diagnostic delay for BMD was approximately 2.7 years, which is about one year longer than that observed in a previous DMD cohort from the same centre [35]. Various factors may contribute to the delay in genetic confirmation of BMD, including sometimes insufficient awareness of the different clinical presentations among primary and/or secondary care clinicians [36]. Studies in DMD suggest that a delay in diagnosis can have a negative impact on the advice, support, and care patients receive both in the shorter and longer term. This includes access to genetic counselling, delayed initiation of available treatments, and preclusion of patients from participating in clinical trials of promising innovative drugs and disease modifying treatments [36–38]. No specific pharmacological treatment for skeletal muscle is currently available for BMD. However, it is particularly important that patients have a cardiac assessment soon after diagnosis, regardless of symptoms, to detect subtle changes in myocardial tissue composition or left ventricular dysfunction. Finding any such abnormality confirms that the left ventricular dysfunction will progress if left untreated. Timely therapy can slow the rate of progression of cardiac dysfunction and, therefore, reduce the likelihood of heart failure and sudden death in the longer term. Similarly, unrecognised neuropsychiatric symptoms prevent patients benefiting from appropriate educational support and reaching maximum level of achievement. Finally, delay in the diagnosis of BMD means that opportunities for timely genetic counselling are missed with consequences both for patients and family members [36–39]. For all these reasons, it is critically important to the long-term welfare of families and patients that medical teams at all levels have a better understanding of the various phenotypes of patients with dystrophinopathy. Additionally, as already suggested, CK testing should be considered in any patient presenting with manifestations suggestive of dystrophinopathy, including mild muscle symptoms, neuropsychiatric issues, or unexplained cardiomyopathy.

The widespread uncertainty about the risk–benefit ratio of corticosteroid therapy for patients with BMD was reflected in our cohort [40]. Very few patients had ever been prescribed corticosteroids for muscle strengthening. Corticosteroid use was typically reserved for patients with severe muscle phenotypes. Mean duration of therapy was short, mainly because of the side effects that evolved, particularly behavioural and/or psychiatric manifestations and excessive weight gain. Moreover, the short treatment duration in most patients also points to only limited benefits of corticosteroids on motor function in BMD. The perceived relative lack of efficacy and prevalence of common side effects requires further evidence from prospective trials of traditional and more recently developed drugs (Vamorolone, NCT05166109).

Consistent with previous publications on patients with BMD, cardiac involvement was present in about half of the patients in our cohort [11, 20, 32, 33]. The detection of left ventricular dysfunction in two patients by the age of 11 years emphasises the importance of early diagnosis, even in children, and having a detailed baseline cardiac assessment. There was no correlation between age at LoA and presence of cardiac involvement, further supporting the view that there does not seem to be direct correlation between the severity of skeletal muscle involvement and the severity or age of onset of cardiomyopathy in BMD [9].

Severe respiratory impairment appears to be uncommon in patients with BMD [12, 13]. Nevertheless, respiratory dysfunction can occur and should be monitored, particularly at more advanced stages of the disease. A significant association was identified between FVC and LoA, highlighting the close relationship between respiratory and skeletal muscle dysfunction [12, 13]. NIV prescription was rarely required and was prompted by reduced FVC% in only half of the patients. This suggests that other factors and comorbidities may contribute to respiratory impairment in adults with BMD rather than the condition alone [41].

In recent years, more attention has been paid to the role that different dystrophin isoforms may play in neurodevelopment [14, 16, 42, 43]. Neuropsychiatric issues or diagnoses were described either at diagnosis or during the course of BMD in almost half of the patients in our cohort. The literature to date reports notable disparities in the prevalence of autism and ADHD between childhood and adulthood in the general population [44, 45]. These disparities will likely also be found among patients with BMD. Therefore, neuropsychological assessments should be considered for all patients with BMD, including adults [44–47]. We hypothesise that the higher prevalence of psychiatric symptoms or diagnoses in the older population (born before 1/1/1990) may stem from underdiagnosed neuropsychiatric disorders in infancy/childhood and/or underdiagnosis/misdiagnosis in adulthood. This could be explained by changes in the definitions of these disorders, particularly autism and ADHD, since the late 1980s, which may have led to a lack of proper identification in older generations.

It is important to note that the prevalence of reported neuropsychiatric or psychiatric features and diagnoses can vary considerably based on cultural influences, which may explain the discrepancies observed in our cohort compared to those in other recently published multi-centre and country-specific studies [22, 24].

Our study also highlights the limitations in accessing diagnostic services to ensure a timely diagnosis and support. Interestingly, patients with neuropsychiatric issues as their first presentation may have a slower rate of progression of skeletal muscle manifestations. Due to the limited number of affected patients in our cohort and the proportion who had not yet been formally assessed, we could not draw definitive interpretations for this observation. From a diagnostic standpoint, this finding suggests that at least CK levels ought to be measured in all individuals with neuropsychiatric features in infancy, even in the absence of overt skeletal muscle signs/symptoms, to investigate the possibility of dystrophinopathy. Ongoing international natural history studies, specifically focusing on neurocognitive and psychiatric implication of dystrophinopathies (NCT04583917 and NCT04668716), should help define the prevalence of neuropsychiatric features, the care needs of these patients, and clarify current uncertainties, including whether there are specific genotype–phenotype correlations.

## Limitations

The retrospective nature of the study inevitably meant that some information was unavailable and/or missing from the dataset. Additionally, some of the oldest patients presented and began their follow up before the *DMD* gene was even discovered. Care arrangements have changed significantly during the time period over which this dataset was accumulated and as BMD became better understood. There were noticeable differences, for example, in the care that older patients received as compared to those diagnosed more recently. Limited numbers of patients in several sub-group analyses, particularly in specific genotypes, limited the power of the associations observed.

## Conclusion

Our analysis provides a detailed cross-sectional snapshot of the clinical phenotypes of patients with BMD. The findings are intended to provide information about the ambulatory status, respiratory needs, extent of cardiac involvement, and neuropsychiatric manifestations to inform discussions with newly diagnosed patients and conversations about long-term care needs and prognosis. A more nuanced description of disease motor function milestones, as outlined in this paper, can help clinicians communicate prognosis to patients in a clearer, more accessible way. These insights could also be valuable in future real-world data and post-marketing surveillance, where more complex motor outcome measures may not be consistently collected across centres worldwide, and to assess the long-term, clinically meaningful impact of new interventions. Combined with similar types of analyses from other centres, these descriptions should allow the development of standardised guidelines for patients with BMD and contribute to the design of clinical trials of novel interventions and therapies.

## Supplementary Information

Below is the link to the electronic supplementary material.

Supplementary file1 (DOCX 1370 KB)

Supplementary file2 (ODS 7 KB)

## Data Availability

Additional data may be shared by the corresponding author upon reasonable request.
