# Supplementary material for: Characterisation of a large, single-centre cohort of patients with Becker muscular dystrophy to inform standardised care guidelines
Source: J Neurol. 2025 Jun 7;272(7):448. doi: 10.1007/s00415-025-13126-9 (PMC12145297; doi:10.1007/s00415-025-13126-9)

**Supplementary Table 1.** Distribution of *DMD* gene variants within the cohort.

| **Variant** | **n** |
| --- | --- |
| Del of exons 45 to 47 | 67 (20 unrelated, 47 from 15 families) |
| Del of exons 45 to 48 | 20 (11 unrelated, 9 from 3 families) |
| Del of exons 45 to 53 | 11 (4 unrelated, 7 from 3 families) |
| Del of exons 45 to 49 | 7 (2 unrelated, 5 from the same family) |
| Del of exons 45 to 55 | 4 (2 unrelated, 2 from the same family) |
| Del of exons 48 to 51 | 3 (unrelated) |
| Del of exon 48 | 2 (unrelated) |
| Del of exons 49 to 51 | 2 (same family) |
| Del of exon 3 | 2 (unrelated) |
| Del of exons 78 to 79 ^a^ | 2 (same family) |
| Del of exons 43 to 44 | 1 |
| Del of exons 2 to 7 | 1 |
| Del of exons 3 to 5 | 1 |
| Del of exons 8 to 19 | 1 |
| Del of exons 48 to 49 | 1 |
| Del of exons 9 to 41 | 1 |
| Del of exons 13 to 30/41 ^b^ | 1 |
| Dup of exons 13 to 16 | 7 (from 3 families) |
| Dup of exons 10 to 18 | 3 (1 unrelated, 2 from the same family) |
| Dup of exons 3 to 4 | 3 (1 unrelated, 2 from the same family) |
| Dup of exons 14 to 27 | 2 (same family) |
| Dup of exons 52 to 55 | 2 (unrelated) |
| Dup of exons 13 to 29 | 1 |
| Small variants | 18 |

Del: deletion; Dup: duplication; n=number. Small variants include small deletions; intronic, missense, and synonymous variants, predicted to affect the splicing process.

^a^ Dystrophin expression on muscle biopsy (available for one individual) consistent with BMD.

^b^ Multiplex PCR allowed to determine the 5’ endpoint of the deletion (exon 12 was present, while exon 13 was deleted); exon 30 was deleted, while exon 42 was present (3’ endpoint lying between exon 30 and 42). Dystrophin expression consistent with BMD on muscle biopsy.

**Supplementary Figure 1**: Correlation between CK levels and age at which CK was measured.


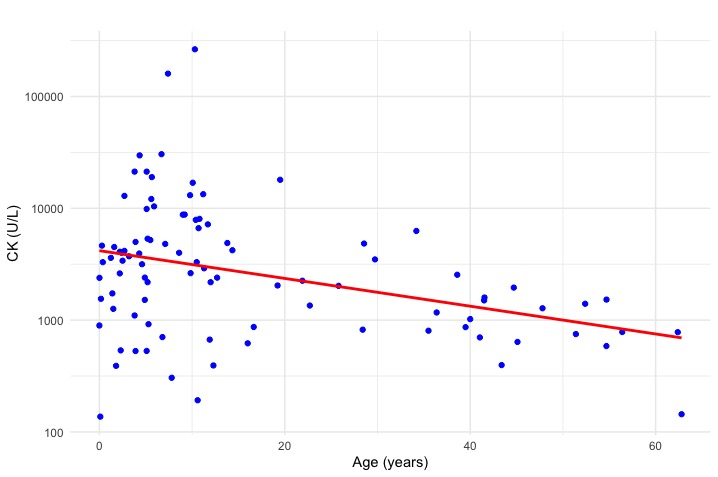

Supplement: Supplementary file 1 — Supplementary file1 (DOCX 1370 KB) [file 415_2025_13126_MOESM1_ESM.docx]
